# Developing Standard Treatment Workflows—way to universal healthcare in India

**DOI:** 10.3389/fpubh.2023.1178160

**Published:** 2023-08-16

**Authors:** Ashoo Grover, Balram Bhargava, Saumya Srivastava, Lokesh Kumar Sharma, Jerin Jose Cherian, Nikhil Tandon, Sudha Chandershekhar, Roderico H. Ofrin, Henk Bekedam, Deepika Pandhi, Aparna Mukherjee, Rupinder Singh Dhaliwal, Manjula Singh, Kavitha Rajshekhar, Sudipto Roy, Reeta Rasaily, Deepika Saraf, Dhiraj Kumar, Neeraj Parmar, Sushil Kumar Kabra, Dhruva Chaudhry, Ashok Deorari, Radhika Tandon, Rajdeep Singh, Binod Khaitan, Sandeep Agrawala, Sudeep Gupta, Satish Chandra Goel, Anil Bhansali, Usha Dutta, Tulika Seth, Neeta Singh, Shally Awasthi, Amlesh Seth, Jeyaraj Pandian, Vivekanand Jha, Sudhanshu Kumar Dwivedi, Reva Tripathi, Alok Thakar, Surinder Jindal, Banglore Nanjudaiah Gangadhar, Anjali Bajaj, Mohan Kant, Aniket Chatterjee

**Affiliations:** ^1^Division of NCD, Indian Council of Medical Research, New Delhi, India; ^2^Department of Cardio Neuro Centre, Indian Council of Medical Research, New Delhi, India; ^3^Division of BMI, Indian Council of Medical Research, New Delhi, India; ^4^Division of ECD, Indian Council of Medical Research, New Delhi, India; ^5^Department of Endocrinology, All India Institute of Medical Sciences, New Delhi, India; ^6^National Health Authority, New Delhi, India; ^7^WHO Country Office for India, New Delhi, India; ^8^Department of Dermatology, University College of Medical Sciences, New Delhi, India; ^9^Indian Council of Medical Research, New Delhi, India; ^10^Department of Paediatrics, AIIMS, New Delhi, India; ^11^Department of Pulmonology, PGIMER, Chandigarh, India; ^12^All India Institute of Medical Sciences, New Delhi, India; ^13^Pandit Bhagwat Dayal Sharma PG Institute of Medical Sciences, Rohtak, India; ^14^Himalayan Institute of Medical Sciences, Baksar Wala, Dehradun, India; ^15^Maulana Azad Medical College, New Delhi, India; ^16^Tata Memorial Hospital, Mumbai, India; ^17^Banaras Hindu University, Varanasi, India; ^18^Post Graduate Institute of Medical Education and Research, Chandigarh, India; ^19^Gastroenterology, Post Graduate Institute of Medical Education and Research, Chandigarh, India; ^20^Haematology, Post Graduate Institute of Medical Education and Research, Chandigarh, India; ^21^Obstetrics and Gynecology, Post Graduate Institute of Medical Education and Research, Chandigarh, India; ^22^Department of Paediatrics, King George's Medical University, Lucknow, India; ^23^Department of Urology, King George's Medical University, Lucknow, India; ^24^Department of Neurology, King George's Medical University, Lucknow, India; ^25^Department of Nephrology, King George's Medical University, Lucknow, India; ^26^Department of Cardiology, King George's Medical University, Lucknow, India; ^27^Department of Obstetrics and Gynecology, King George's Medical University, Lucknow, India; ^28^Department of ENT, King George's Medical University, Lucknow, India; ^29^Department of Pulmonology, King George's Medical University, Lucknow, India; ^30^Department of Psychiatry, King George's Medical University, Lucknow, India; ^31^Department of Gastroenterology, King George's Medical University, Lucknow, India; ^32^Christian Medical College, Ludhiana, India; ^33^The George Institute for Global Health, New Delhi, India; ^34^King George's Medical University, Lucknow, India; ^35^National Institute of Mental Health and Neurosciences, Bengaluru, India; ^36^Government of Himachal Pradesh, Himachal Pradesh, India; ^37^Department of Paediatrics, Indian Council of Medical Research, New Delhi, India; ^38^Department of Opthalmology, Indian Council of Medical Research, New Delhi, India; ^39^Department of General Surgery, Indian Council of Medical Research, New Delhi, India; ^40^Dermatology, Indian Council of Medical Research, New Delhi, India; ^41^Paediatric Surgery, Indian Council of Medical Research, New Delhi, India; ^42^Oncology, Indian Council of Medical Research, New Delhi, India; ^43^Orthopaedics, Indian Council of Medical Research, New Delhi, India; ^44^Endocrinology, Indian Council of Medical Research, New Delhi, India

**Keywords:** Standard Treatment Workflows (STWs), universal health coverage (UHC), quality health care (QHC), public health, disease

## Abstract

Primary healthcare caters to nearly 70% of the population in India and provides treatment for approximately 80–90% of common conditions. To achieve universal health coverage (UHC), the Indian healthcare system is gearing up by initiating several schemes such as National Health Protection Scheme, Ayushman Bharat, Nutrition Supplementation Schemes, and Inderdhanush Schemes. The healthcare delivery system is facing challenges such as irrational use of medicines, over- and under-diagnosis, high out-of-pocket expenditure, lack of targeted attention to preventive and promotive health services, and poor referral mechanisms. Healthcare providers are unable to keep pace with the volume of growing new scientific evidence and rising healthcare costs as the literature is not published at the same pace. In addition, there is a lack of common standard treatment guidelines, workflows, and reference manuals from the Government of India. Indian Council of Medical Research in collaboration with the National Health Authority, Govt. of India, and the WHO India country office has developed Standard Treatment Workflows (STWs) with the objective to be utilized at various levels of healthcare starting from primary to tertiary level care. A systematic approach was adopted to formulate the STWs. An advisory committee was constituted for planning and oversight of the process. Specialty experts' group for each specialty comprised of clinicians working at government and private medical colleges and hospitals. The expert groups prioritized the topics through extensive literature searches and meeting with different stakeholders. Then, the contents of each STW were finalized in the form of single-pager infographics. These STWs were further reviewed by an editorial committee before publication. Presently, 125 STWs pertaining to 23 specialties have been developed. It needs to be ensured that STWs are implemented effectively at all levels and ensure quality healthcare at an affordable cost as part of UHC.

## 1. Introduction

Having sound health is a public right, and providing quality healthcare to its citizens is the joint responsibility of every government and individual. India's population as of 2023 is estimated to be 1.425 billion. This is a key challenge. Delivering healthcare through primary health is an approach to designing and delivering health services that lay the foundation for achieving universal health coverage (UHC). UHC means that each individual has access to health services whenever they need and without any financial hardships. The three-pronged dimensions of UHC cater to quality health services, robust financial management, and assurance of equity and ease of access for its population (1, 2).

Quality healthcare aims to be safe, effective, patient-centered, timely, equitable, and efficient (3) To achieve UHC for providing quality health services, one of the components is that healthcare providers should be well versed with the simplified protocols of disease management (4, 5).

For ensuring optimum management, the primary, secondary, and tertiary care physicians in the public or private sector need to be oriented in a regular fashion through trainings, workshops, self-reading, and social media with updates of the manuals, latest management guidelines, and protocols that are developed by research and academic institutions/national and international associations. The physician's updated knowledge has implications for improving patient outcomes, which implies the need for enhanced clinical competence (6, 7). To further enhance the competence, it is also important to re-orient the newly inducted physicians to broader, comprehensive, and simplified clinical pathways/algorithms which cover the common and specific disease conditions so that the patients at any level (rural, urban/primary, secondary, and tertiary) can be treated in a satisfactory manner with the assurance of a minimum quality of care.

A treating physician must document a holistic concept while managing the patient, wherein the nature, duration of illness, preventive measures, appropriate drugs, duration of therapy, follow-up, drug precautions, side effects, and plans to refer and evaluate are informed to the patients (6, 8). To facilitate this approach in daily practice, a comprehensive guiding document should be available, wherein all the aspects are covered in a simplified approach.

Different methods of depicting management process. A guideline is a statement that determines a course of action. A guideline aims to streamline particular processes according to a set routine or sound practice. Standard Treatment guidelines are systematically developed documents to facilitate the physicians for providing better and appropriate healthcare. Treatment algorithms are clear, authoritative, and concise treatment pathways for medical conditions to guide healthcare professionals in their decision-making when planning, executing, and evaluating care. They direct both assessment and management of a clinical problem and define the endpoint of the decision-making process. Workflows are defined as a set of tasks that are grouped chronologically into processes. Clinical workflow is aimed at improving the functionality of the healthcare system with the ultimate purpose of streamlining the process and offering patients the best health experience possible (9). Delivering integrated primary healthcare requires practices to be followed that enable patient identification, engagement, treatment, and monitoring/adjusting care for achieving uniformity, equity, and a minimum standard of care, and for this, some simplified documents in the form of workflows would be the suitable approach.

Measuring the quality of the process of delivering healthcare and the resulting health outcomes is especially challenging, requiring methods and approaches that go beyond standard service statistics and facility surveys.

Standard Treatment Guidelines (STGs), standard treatment protocols or therapeutic guidelines, are systematically developed statements that are designed to assist practitioners and patients in making informed decisions about suitable healthcare for specific clinical conditions. The basic purpose of the STGs is to guide clinicians, pharmaceutical personnel, and all other health professionals, to use medicines rationally for the benefit of the patients. These play a critical role in ensuring evidence-based clinical practice and quality of care. At the health system level, it helps in the planning and costing of services. Standards Treatment Guidelines also become an important tool for monitoring and authorizing procedure in publicly funded health insurance schemes. With these quality control, regulatory, and planning functions, standard treatment guidelines framed by experts become indispensable tools both for public and private service providers and healthcare cost analysis (7).

As toward attainment of UHC, the Indian healthcare system is gearing up by initiating several schemes such as the National Health Protection Scheme, Ayushman Bharat, Nutrition Supplementation Schemes, and Inderdhanush Schemes; the healthcare delivery system is facing challenges such as irrational use of medicines, over- and under-diagnosis, high out-of-pocket expenditure, lack of targeted attention to preventive and promotive health services, and poor referral mechanism. Some of the other challenges, such as low evidence of therapeutic use of drugs, less reliable reference sources for treatment guidelines, and lack of updated knowledge, perpetuate the problem.

With the volume of growing new scientific evidence and rising healthcare costs, healthcare providers are not able to keep pace with bulky voluminous treatment guidelines and reference manuals from diverse sources. There needs to be a more simplified approach to be adopted by physicians which can guide them in the appropriate management of patients whose numbers are increasing every day. The doctor–patient ratio is 1:1000 and is rapidly increasing in terms of the number of patients; thereby, the time spent per patient is less. Physicians definitely require a simplified updated approach in the form of a one-pager, app-based tool, which can continuously guide the treating physicians as they manage the patient and can manage a large number of patients in a short time span and with rationale treatment.

Therefore, there are various standard treatment guidelines and processes by different organizations, but they are very voluminous, complex, and not so easy to refer to. In this context, it is imperative to state that physicians require some handy, simple, and easy-to-refer-to guiding document, which can be accessible in multiple ways. The workflows are the ones that can have such characteristics. Thus the Standard Treatment Workflows aims to empower all levels of healthcare physicians so that they can come up with quick decision-making simple management.

This study describes the detailed process of developing Standard Treatment Workflows to be used by physicians, at various levels of healthcare starting from primary to tertiary level care. These are made available by the Government of India to be adopted and followed by all providers. The salient features of the workflows are that these are simple to use, one pager, easy to follow, mention commonly encountered signs and symptoms, emphasize on highest efficacy, and high safety drugs with the least adverse events. Depiction through images has provided more clarity to facilitate the diagnosis; hence, these workflows have clear visual clues with simple text.

## 2. Methodology and analysis

### 2.1. Constitution of advisory committee

A stratified approach has been taken to formulate the Standard Treatment Workflows through partnering with the National Health Authority Govt. of India and the WHO representative office, India, to align the priority areas of STWs with national health priorities. An advisory committee was constituted with representatives from the national-level institutes such as the National Institute for Transforming India (NITI) Aayog, the National Health Systems Resource Center, WHO, the Department of Health & Family Welfare, academic and research institutions in public as well as private set up, and various associations. The advisory committee deliberates in detail about the topics, on which conditions the STWs have to be developed and these are prioritized based on the disease dynamics.

### 2.2. Topic prioritization

Prioritization of the topic was one of the key tasks as there was a need to choose such topics that are common in India and useful for physicians. The topic selection was made by the respective domain-specific expert members, and it was based on criteria. The criteria include the burden of disease, commonality of the condition at the primary health center, availability of resources at the facility, and acute or chronic conditions. After topic selection, approval was taken from the advisory committee, and further meetings were conducted with the domain specialty experts and the relevant government agencies.

### 2.3. STW framework and sections

The STW framework and layout were prepared. It was decided to have a one-pager document for each condition and would have the clinical presentation and diagnostic investigations, which were thought to be kept as essential, desirable, and optional so that unnecessary investigations could be avoided. Sometimes, these unnecessary investigations are the main reasons for out-of-pocket expenditure (OOP) along with the increasing burden of disease (10). The management section is to have it at all levels of the healthcare system, i.e., primary, secondary, and tertiary. The one-page document of STW also contained some sections like do's and don'ts that will guide the physicians, so that untoward happenings can be avoided. The management of outdoor and indoor patients also has Been mentioned in the STWs. The pictorial images of the specific conditions have also been depicted. When to refer is also one of the features in STWs which can help the physicians when to refer a patient to a higher facility either due to a resource crunch or needing specialized services. The situation requires emergency and immediate management have been depicted with red flag warning signs for quick management and referral. The disclaimer section in STW is articulated in a manner that is not a binding to the treating physician and has been developed in consultation with the national-level experts. Each STW has a section on abbreviations and references as well in a unified way.

### 2.4. Developing STW through national-level experts

**Figure 1:**
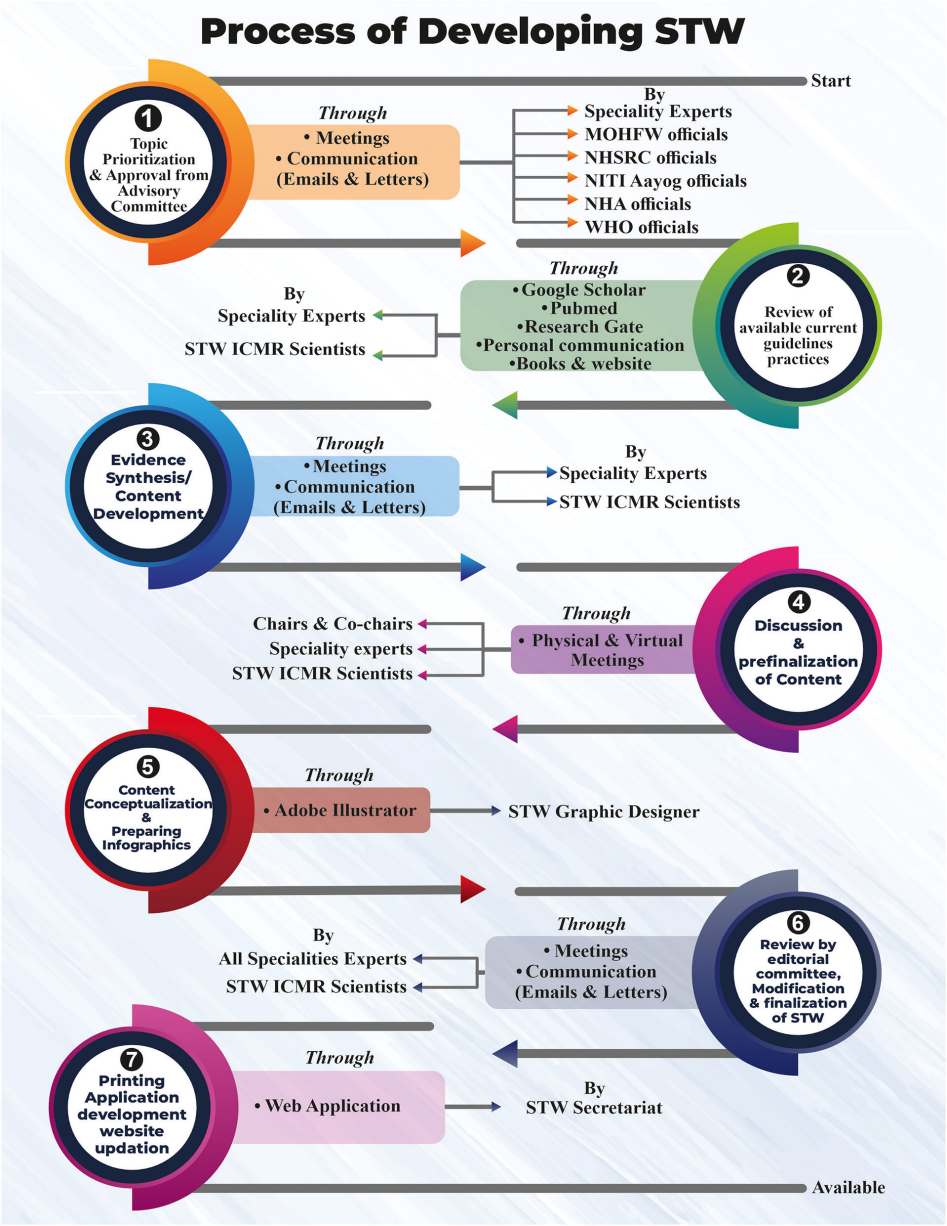
Process of developing STW.

The Standard Treatment Workflows have been developed by national-level experts constituted for each discipline separately. The experts have been drawn from different institutions who have vast and long experience in working in their field and seeing large volumes of indoor/outdoor patients. Experts were explained about the purpose of the STWs being developed, they subsequently deliberated on the content of the STW and discussed the features/matter which had to be included in a one-pager document. The images of the conditions for diagnostic findings were also decided to be inserted at a suitable place. To maintain uniformity throughout all STWs, the different sections of the STW were discussed individually and then finalized. Once the content was finalized, the infographics were prepared in standard software, which was also reviewed by specialty experts (Figure 1). During the expert's deliberations, the topics were discussed and the addition of conditions to be included was done.

Each STW was further reviewed to ensure the clarity of communicating an appropriate message and uniformity through internal editorial and external editorial committees for proofreading, managing editorial correction, and monitoring the quality of the STWs. Each STW revision was time-consuming to look for the aspects of uniformity, clarity, grammar, due credits for intellectual property, and alignment with the existing guidelines. To make this editing more transparent and comprehensive, the STW of one specialty was reviewed by experts of other specialties. This could avoid the conflict of interest and improve the quality of particular STW. The experts developing the STW and the experts editing the STW did the systematic analysis to evaluate clinical practice at all three levels.

The revisions of each STW were undertaken in consultation with experts through email, telecommunications, and occasionally one-to-one physical meetings. After editorial corrections, the STWs were sent to Advisory Committee members for finalization before printing/publishing. During the whole process of preparing the STWs, the consultation with the National Health Authority, State Health Departments, and WHO India Country Office was done from time to time at all steps.

At the end of the document, the disclaimer has been mentioned in each STW. Each STW is prepared considering the evidence, best practices, and expert recommendations. These are prepared with an emphasis on the limitations in the availability of resources in various Indian healthcare settings. These are primarily meant for physicians to guide their practice and cannot be used for legal purposes. To establish linkages between the STWs, reference has been provided as required and all STWs have been ICD 10 code.

### 2.5. Number of STW published

To date, STWs in 122 conditions covering 23 disciplines have been published (Table 1); in three volumes, volume 1 of STWs for 53 conditions in 9 specialties, a special edition volume 2 of STWs in Tuberculosis covering 3 specialties with 18 STWs, and volume 3 for 51 conditions in 11 specialties have been published (Table 2).

**Table 1:** Number of specialties and conditions.

**Volume**	**Specialties**	**Conditions**
1	9	53
2	3	18
3	11	54
Total	23	125

**Table 2:** Condition-wise details of STW.

**Conditions**	**STW**	**Conditions**	**STW**
Cardiology	6	Dermatology	14
ENT	7	Endocrinology	6
Nephrology	4	Gastroenterology	4
Neurology	6	General Surgery	5
OBG	6	Neonatology	10
Pediatric	7	Oncology	3
Psychiatry	8	Ophthalmology	3
Pulmonology	4	Orthopedics	2
Urology	5	Pediatric Surgery	5
Extra Pulmonary Adult TB	10	Infertility	1
Pediatric Pulmonary TB	5	Genetic Disorders	1
Invx. & Rx TB	3		

### 2.6. Availability of STW

To facilitate access and increase use, the Standard Treatment Workflows have been published in different formats that include hardbound copies, posters, e-version on Govt. websites/web portals https://stw.icmr.org.in and https://nha.gov.in, and a mobile application downloadable on Android and iOS platform.

### 2.7. Experiences of experts while developing STW

**Figure 2:**
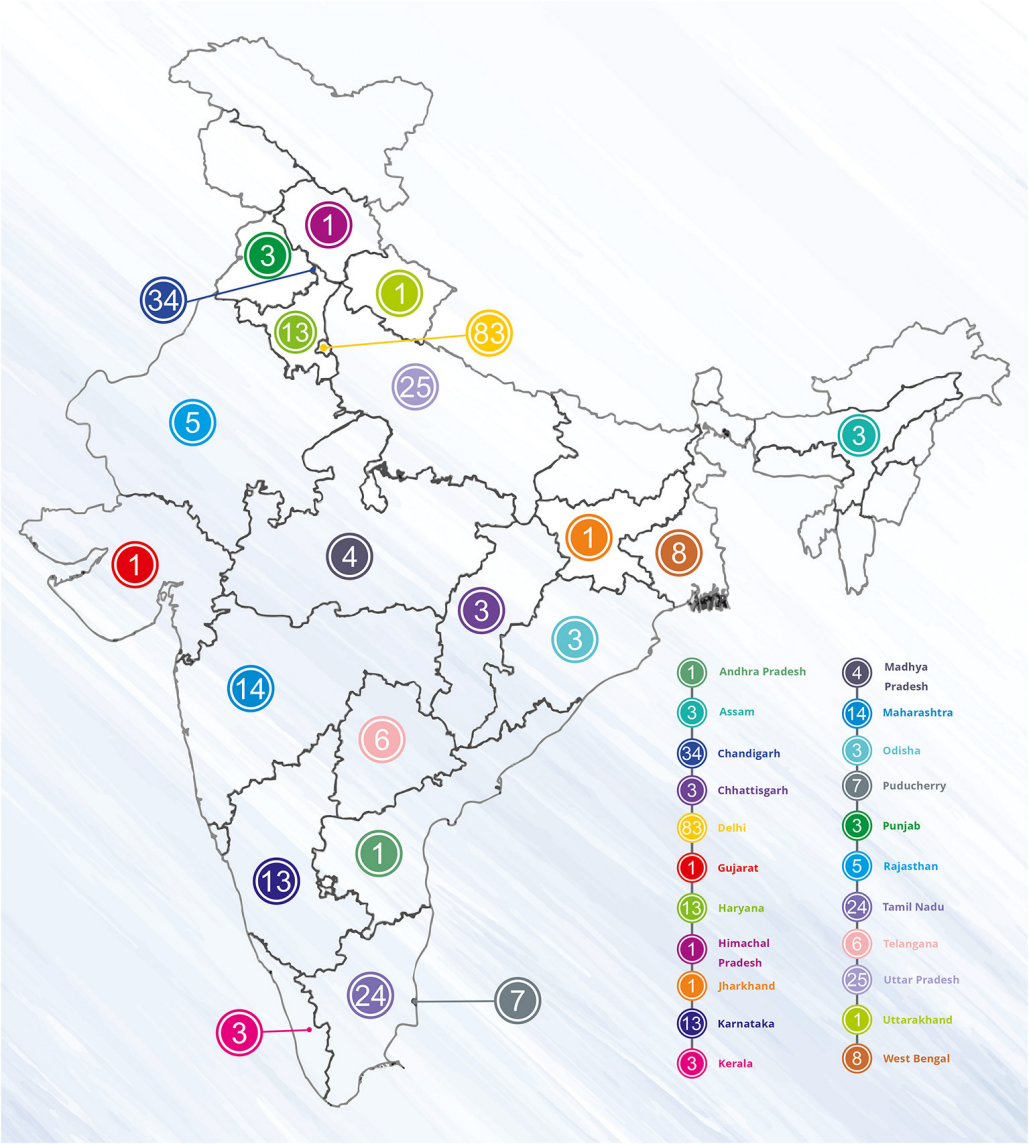
State-wise distribution of experts.

The experts from all over the country (Figure 2) discussed this at length while developing STWs to fit the content on one page. Putting across the lengthy guidelines on one page was a challenging task especially when everything about that condition was needed to be included, as per experts “gagar mein sagar jisko kehte hain, bahut mushkil tha” which means “it was a difficult task to express too much in limited words and to convey the right meaning”. Another challenge was the selection of topics; it had to be a common condition, which is encountered at all levels of healthcare. Experts also faced the challenge as the user could be an undergraduate, postgraduate, experienced physician, or even a private practitioner, so the content has to be presented in simple language and updated. As per experts, the principle followed was “collect material, then edit edit edit, shorten shorten shorten”.

The majority (40%) of the experts had an experience of more than 15 years of treating patients in their respective discipline (maximum = 34 years), and 94% of the experts had more than 100 publications in the related discipline (maximum = 645).

### 2.8. Future work

The task of developing Standard Treatment Workflows is a continuous process with wider scope and implications. With support from the present leadership in the Ministry of Health and Family Welfare, Govt. of India, for developing these Standard Treatment Workflows, there is a need to continue this study for other conditions. However, there is a request to make them simpler for use by clinicians and also to be specific in identifying which level of care this STW is meant for. Furthermore, the concurrent monitoring and evaluation of the Standard Treatment Workflows has been suggested by various experts and academic institutions. Henceforth, in addition to preparing new STWs, the concurrent evaluation of these workflows is being proposed in different levels of setting. To date, through an interactive meeting with 20 states, dissemination of STWs has been done and it has been presented at national and international conferences as well. There is also a need to create a feedback loop so that comments and suggestions from users can be reviewed and discussed to improve the existing STWs.

### 2.9. Way forward

Many physicians treat common conditions in their own way, leading to variations in treatment patterns. Physicians at the primary healthcare level who have to see many patients of almost every condition need these simplified approaches; these STWs will be very useful for them. Treating the patients at super-specialty facilities, where all the departments are fully equipped is much easier and more appropriate, almost all the investigations can be performed than managing the patients at primary healthcare facilities, which are resource deficient, and proper diagnosis for a few conditions cannot be made. The cases such as febrile seizures, drug-induced toxic epidermolysis, and sudden blindness due to glaucoma need an early referral from primary healthcare and that is possible only if the physicians at the PHC level are able to identify these. These STWs are a resource to them.

For the first time, a one-pager document, i.e., STWs, has been developed in India and is getting wider popularity. These are being disseminated through different conferences, state-level meetings, and sharing the apps. It is also proposed to undertake implementation/operational research to find out the utility, relevance, and adoption challenges at select study sites.

Effective implementation, however, is perhaps the greatest challenge in introducing STGs. When implemented effectively, it offers several advantages to patients, healthcare providers, supply management personnel, and policymakers.

Many states and authorities in India have been developing different STGs which are not available online. There is a need to ensure that STGs are made freely available online which could help in wider adoption in clinical practice (11).

The future study on STW would remain continued, and more robust mechanisms would be in place such as the topic selection as per the felt need from clinicians at all levels, the burden of disease, and the mode of utilization. Implementation research would be the next step for its constant monitoring and evaluation as well as constant updating. National Health Authority (NHA), a partner agency with ICMR STW, is implementing and disseminating these STWs through the Ayushman Bharat Scheme (ABS) and Ayushman Bharat Digital India (ABDI) in various state-level and district-level meetings. A joint research study with NHA and WR India Office would further generate evidence for its wider scope to reach its end user.

## Data availability statement

The datasets presented in this study can be found in online repositories. The names of the repository/repositories and accession number(s) can be found below: https://stw.icmr.org.in/stws-download.

## Author contributions

AG, BB, SS, and LS conceptualized the study. AG, SS, and LS drafted the manuscript. JC, DP, AM, RD, SR, DK, and NP helped with redrafting for submission. NT, SC, RO, HB, MS, KR, RR, DS, SK, DC, AD, RTa, RS, BK, SAg, SG, SCG, ABh, UD, TS, NS, SAw, AS JP, VJ, SD, RTr, AT, SJ, and BG provided subject matter expertise. All authors read and approved the final manuscript submission and contributed substantively to this study.
